# A multi-country analysis of the prevalence and factors associated with bullying victimisation among in-school adolescents in sub-Saharan Africa: evidence from the global school-based health survey

**DOI:** 10.1186/s12888-021-03337-5

**Published:** 2021-07-01

**Authors:** Richard Gyan Aboagye, Abdul-Aziz Seidu, John Elvis Hagan, James Boadu Frimpong, Eugene Budu, Collins Adu, Raymond K. Ayilu, Bright Opoku Ahinkorah

**Affiliations:** 1 Department of Family and Community Health, School of Public Health, University of Health and Allied Sciences, PMB 31, Ho, Ghana; 2 Department of Population and Health, University of Cape Coast, Cape Coast, Ghana; 3 College of Public Health, Medical and Veterinary Services, James Cook University, Townsville, Australia; 4 Department of Estate Management, Takoradi Technical University, Takoradi, Ghana; 5 Department of Health, Physical Education, and Recreation, University of Cape Coast, Cape Coast, Ghana; 6 Neurocognition and Action-Biomechanics-Research Group, Faculty of Psychology and Sport Sciences, Bielefeld University, Bielefeld, Germany; 7 Department of Health Promotion, Education and Disability Studies, Kwame Nkrumah University of Science and Technology, Kumasi, Ghana; 8 Faculty of Arts and Social Sciences, University of Technology Sydney, Sydney, Australia; 9 School of Public Health, Faculty of Health, University of Technology Sydney, Sydney, Australia

**Keywords:** Abuse, Bullying, In-school adolescents, Physical harm, Sub-Saharan Africa, Victimisation

## Abstract

**Background:**

Over the past few years, there has been growing public and research interest in adolescents’ experiences with various forms of bullying victimisation because of their psychological, emotional, and/ or physical consequences. The present study examined the prevalence of bullying victimisation and its associated factors among in-school adolescents in sub-Saharan Africa.

**Methods:**

Using data from the Global School-based Health Survey (GSHS) from 2010 to 2017 of eleven sub-Saharan African countries, a sample of 25,454 in-school adolescents was used for analysis. Statistical analyses included frequencies, percentages, Pearson chi-square and multivariable logistic regression. Results were presented as adjusted odds ratios (aOR) at 95% confidence intervals (CIs).

**Results:**

The overall prevalence of bullying victimisation among the respondents was 38.8%. The prevalence was lowest in Mauritius (22.2%) and highest in Sierra Leone (54.6%). Adolescents who felt lonely [aOR = 1.66, 95% CI = 1.53, 1.80], had history of anxiety [aOR = 1.53, 95% CI = 1.41, 1.66], suicidal ideation [aOR = 1.28, 95% CI = 1.17, 1.39], suicidal attempt [aOR = 1.86, 95% CI = 1.72, 2.02], current users of marijuana [aOR = 1.59, 95% CI = 1.38, 1.84], and truants at [aOR = 1.43, 95% CI = 1.34, 1.52] were more likely to be victims of bullying. Conversely, adolescents who had peer support were less likely to be victims of bullying [aOR = 0.78, 95% CI = 0.73, 0.82]. Adolescents aged 15 years or older had lower odds of experiencing bullying victimization compared to their counterparts aged 14 years or younger [aOR = 0.74, 95% CI = 0.69, 0.78].

**Conclusion:**

Our findings suggest that age, loneliness, anxiety, suicidal ideation, suicidal attempt, and current use of marijuana are associated with increased risk of bullying victimisation. School-wide preventative interventions (e.g., positive behavioural strategies- Rational Emotive Behavioral Education, [REBE], peer educator network systems, face-face counseling sessions, substance use cessation therapy) are essential in promoting a positive school climate and reduce students’ bullying victimisation behaviours.

**Supplementary Information:**

The online version contains supplementary material available at 10.1186/s12888-021-03337-5.

## Background

One common form of violence often found within the school context among children and youths is bullying [1]. Hence, bullying victimisation among in-school adolescents remains a serious concern because of its link with a host of mental health- anxiety disorder, depression; physical health- injuries, and academic problems- adjustment, low achievement across many countries worldwide [2, 3]. Bullying is defined as a harsh or aggressive behaviour directed at the victim by a perpetrator with the intent of causing psychological, emotional or physical harm as a result of an imbalance of power [4] or aggressive, intentional acts carried out by a group or an individual repeatedly and overtime against a victim who cannot easily defend him or herself [5]. Despite some debate over the definition, many scholars posit that bullying encompasses the resolve to inflict harm and demonstrating an imbalance of power between the aggressor and the victim, which happens recurrently [5, 6]. The imbalance of power commonly manifests from physical strength, social status in the group, or from group size (e.g., a group targeting a single person) by recognising a person’s vulnerabilities (e.g., appearance, learning problem, family situation, personal characteristics) and using this information to harm him or her [1]. Evidence suggest that bullying ranges from verbal attacks (e.g., name-calling, threats), physical behaviours (e.g., hitting, kicking, damaging victim’s property), and relational/social aggression (e.g., social exclusion, rumour spreading [5, 7, 8]) to current forms of abuse via internet and emerging technologies, popularly termed as cyberbullying.

There seems to be a wide disparity in the estimation of bullying victimisation across studies and reports, partly because of variations in measurement and/or conceptualisation of the bullying construct. Besides, other context-specific (e.g., linguistic variations) and socio-cultural (e.g., masculinity-feminity) determinants across different societies may determine who does the bullying (e.g., friends in the same class or strangers), where it happens (e.g., classroom, playground), and types of bullying (e.g., social exclusion, extortion, physical abuse, [1]). Recent studies [9, 10] reveal that nearly 20–25% of youth are connected with bullying as culprits, victims, or both. For Western societies, approximately 4–9% of youths regularly engage in bullying behaviours, with 9–25% of school-age children being bullied. Although more studies have been done on bullying victimisation across Western and Eastern high-income countries, limited research attention has been given to the same constructs in low- and middle-income countries [10].

Existing scholarly evidence from sub-Saharan African countries shows that Ghana, Mozambique, Nigeria and Malawi have reported a high prevalence of bullying victimisation (i.e., 56% [11], 45.5% [3], 16.3% [12], and 44.5% [13]) respectively. Additionally, socio-demographic (e.g., sex, age, economic status: [3, 12–18]) and behavioural characteristics (e.g., loneliness, physical fighting, sexual behaviours, substance use, truancy: [2, 3, 19–21]) have been identified as correlates of bullying victimisation. Specifically, bullying victimisation is inclined towards boys [22, 23]; less prevalent with increasing age [22, 24, 25]; increaseswith loneliness [26]; high among those who have no friends [27]; high among thosw who always have negative feelings, such as worries, sadness, unhappiness, or hopelessness [28]; high among those with low self-esteem [29]; and those suicide ideation [26].

Apart from the variations in prevalence estimates cited earlier because of varied conceptualisations and measurements used, research on sub-populations of bullying victimisation is relatively sparse [30]. Moreso, only a few studies have used large sample data for stable prevalence estimation and cross-country comparisons [30]. The lack of conclusiveness on bullying victimisation may partially also be ascribed to research not adequately addressing other multidimensionality and cross-contextuality of bullying [31]. For instance, school location (i.e., rural-urban setting) and socio-economic classification (i.e., elite-non-elite) may be important correlates of bullying victimisation. Most schools in sub-Saharan Africa (SSA) are typically categorised according to rural-urban, public-private and elite-non-elite strata, such that considerable public resources and funding are allocated unfairly according to these classifications. Unequal opportunities and resources for education in different types of geographical areas can worsen the already socio-economic disparities that negatively impact on academic achievements of students and thus may predict different patterns of bullying victimisation [32, 33].

Even though few country-specific studies have been conducted in some countries in SSA [3, 11–13], no available study has examined the prevalence and predictors of bullying victimisation among in-school adolescents in SSA using nationally representative data. The limited number of localised research is often beset with the unrepresentative and/ or unreliable datasets with small samples; hence findings have often been exaggerated with limited generalizability to guide public health action [33]. Therefore, this current study examined the prevalence and predictors of bullying victimisation among in-school adolescents in SSA using data from the Global School-based Health Survey (GSHS) in eleven countries in SSA. Current findings would play a vital role in the design and implementation of anti-bullying policies and strategies, as well as strengthen the existing ones to curb bullying victimisation in schools across selected the sub-Saharan African countries.

## Methods

### Data source and study design

The study involved secondary data analyses of the GSHS dataset of eleven countries in SSA between 2010 and 2017. The GSHS is a school-based survey that uses self-administered questionnaires to collect data on adolescents’ health behaviours and protective factors related to the leading causes of morbidity and mortality. The health behaviour and protective factors the GSHS questionnaire measures include; alcohol and other drug use, dietary behaviours, hygiene, mental health, physical activity, protective factors, sexual behaviour, tobacco use, violence, and unintentional injury. The survey is conducted in low-income and middle-income countries and is coordinated by the health and educational ministries in various countries with technical support from the World Health Organization (WHO) and the Centre for Disease Control and Prevention (CDC). A cross-sectional study design was used in collecting data from in-school adolescents. The questionnaire used contained closed-ended questions. The questionnaire used in this study has previously been published elsewhere (See Table S1). The dataset to the various surveys are freely available online and has been provided as a supplementary file 1 (Table S1). The GSHS data were collected from a nationally representative sample of students. Students who met the inclusion criteria and provided evidence of written informed consent were given questionnaires to complete. The students provided their responses on a computer scannable form distributed by trained staff during a class period. We relied on the “Strengthening the Reporting of Observational Studies in Epidemiology” (STROBE) statement for writing the manuscript.

### Sampling technique

A two-stage cluster sampling technique was used to select schools and students for inclusion in the study. First, schools were randomly selected based on probability proportionate to the school’s enrolment size. Secondly, the classes were selected randomly, and all students in the selected class who met the eligibility criteria were recruited for inclusion into the study. The sampling technique employed ensured that every student had an equal chance of participating in the study. The sampling process was the same in all participating countries. A total of 25,454 in-school adolescents with complete cases on the variables of interest were included in the study. The sample distribution and prevalence of bullying victimisation is shown in Table 1.

**Table 1:** Sample distribution and prevalence of bullying victimisation among the in-school adolescents in sub-Saharan Africa

Country	Year of publication	Population	Sample^a^	Sample^b^	Prevalence of bullying victimization
Benin	2016	2536	2219	911	41.1
Eswatini	2013	3680	2944	877	29.8
Ghana	2012	3632	2821	1391	49.3
Liberia	2017	2744	1499	667	44.5
Mauritania	2010	2063	1531	668	43.6
Mauritius	2017	3012	2491	553	22.2
Mozambique	2015	1918	1319	563	42.7
Namibia	2013	4531	3525	1534	43.5
Seychelles	2015	2540	1891	822	43.5
Sierra Leone	2017	2798	2118	1156	54.6
Tanzania	2014	3793	3096	733	23.7
Total		**33,247**	**25,454**	**9875**	**38.8**

Sample^a^ = Sample with complete cases of variables used in the study; Sample^b^ = Number of respondents who were bullied in the past 30 days prior to the data collection

### Study variables

#### Outcome variable

The main outcome variable in the study was bullying victimisation. Bullying victimisation was derived from the question “During the past 30 days, on how many days were you bullied?”. The responses ranged from 1 = 0 days to 7 = All 30 days. Responses were dichotomised into Yes and No. The students who responded “0 days” were categorised as not bullied (No) and those who reported at least 1 day were grouped as being bullied (Yes). This categorisation was informed by previous studies [2, 34].

#### Explanatory variables

The explanatory variables were selected based on their availability in the GSHS dataset as well as the significant associations between these variables and bullying victimization as established in previous studies [2, 3, 34]. These variables include age, sex, truancy, marijuana use, peer support, close friends, suicidal ideation, suicidal attempt, loneliness, anxiety, parental or guardian supervision, parental or guardian bonding, and parental or guardian connectedness. The detailed description of the variables and the recoded responses have been shown in the supplementary file 2 (Table S2).

### Statistical analyses

The data analysis was performed using Stata software version 16.0 (Stata Corporation, College Station, TX, USA). The datasets were extracted, cleaned, recoded, and appended into one dataset of the country- as the name identifies. The analysis was carried out in three stages. First, the prevalence of bullying victimisation in all the countries was presented in a tabular form (Table 1). At the second stage, a bivariate analysis was carried out to determine the prevalence of bullying victimisation across the explanatory variables. Their respective *p*-values were determined using a Pearson Chi-square test. All the variables that showed significance had *p* < 0.05 were included in the third model (multivariable analysis). For the third stage, a multivariable regression analysis employing a binary regression model was performed. The binary regression model was used because the outcome variable was dichotomised into binary form (Yes/No),which enabled our data to meet the underlying assumption for the analysis. Two regression models (Model I and II) were used in the study. Model I consisted of all the explanatory variables that were significant from the chi-square analysis and bullying victimization. In model II, the analysis was adjusted by adding the countries to assess the strength of the association between the explanatory variables and bullying victimisation while controlling for countries. The results of the regression analyses were presented using the adjusted odds ratio (aOR) and their respective 95% confidence interval (CIs), signifying the level of precision. Statistical significance was set at *p*-value < 0.05 or 5% in all the analyses. All the recoded variables and reference categories used in the study were based on findings from previous studies that used the GSHS dataset [2, 35]. A multicollinearity test using the Variance Inflation Factor (VIF) was carried out to check for any possibility of correlation among the explanatory variables. The result showed that the mean VIF was 1.53; hence, no evidence of collinearity.

### Ethical consideration

The survey was conducted with strict adherence to ethical protocols. In various countries, institutional permission was sought from either the Ministry of Education or the Ministry of Health. All the ethical requirements from these institutions were strictly adhered to, especially, concerning the inclusion of minors in a study. At the school level, written informed consent was sought from the heads of various schools included in the study. For adolescents below 18 years, parental or guardian consent and child assent were sought from them before inclusion into the study. Also, written informed consent was obtained from those aged 18 years or older. The sampled students anonymously and voluntarily completed the survey questionnaire.

## Results

### Prevalence of bullying victimisation among the in-school adolescents in SSA

Results from Table 1 show that the overall prevalence of bullying victimisation among the respondents was 38.8%. The prevalence was lowest in Mauritius (22.2%) and highest in Sierra Leone (54.6%).

### Bivariate analysis of bullying victimisation across the background characteristics of the adolescents in SSA

Table 2 shows the results of the bivariate analysis of bullying and background characteristics. The results revealed that age (χ^2^ = 7.60, *p* = 0.006), loneliness(χ^2^ = 469.28, *p* = < 0.001), anxiety (χ^2^ = 422.07, *p* = 0.000), suicidal ideation (χ^2^ = 438.64, *p* = < 0.001), suicidal plan (χ^2^ = 397.41, *p* < 0.001), suicidal attempt (χ^2^ = 865.34, p = 0.000), current marijuana use (χ^2^ = 170.44, *p* = < 0.001), truancy (χ^2^ = 391.59, *p* < 0.001), peer support (χ^2^ = 134.46, *p* < 0.001), parental or guardian supervision (χ^2^ = 26.28, *p* < 0.001), parental or guardian connectedness (χ^2^ = 108.60, *p* = < 0.001), and parental or guardian bonding (χ^2^ = 62.14, *p* = < 0.001) were statistically associated with bullying victimization among the respondents.

**Table 2:** Bivariate analysis of bullying victimisation across the background characteristics of the in-school adolescents in sub-Saharan Africa

Variables	*N* = 25,454		Bullying victimization		Chi-square (*p*-value)
	Frequency	Percentage	No (%)	Yes (%)	
Age					7.60 (0.006)
14 years or younger	8222	32.3	60.0	40.0	
15 years or older	17,232	67.7	61.8	38.2	
Sex					0.17 (0.676)
Female	13,153	51.7	61.1	38.9	
Male	12,301	48.3	61.3	38.7	
Felt lonely					469.28 (< 0.001)
No	22,273	87.5	63.7	36.3	
Yes	3181	12.5	43.7	56.3	
Anxiety					422.07 (< 0.001)
No	22,354	87.8	63.5	36.5	
Yes	3100	12.2	44.4	55.6	
Suicidal ideation					438.64 (< 0.001)
No	21,323	83.8	64.0	36.0	
Yes	4131	16.2	46.7	53.3	
Suicidal plan					397.41 (< 0.001)
No	20,904	82.1	64.0	36.0	
Yes	4550	17.9	48.2	51.8	
Suicidal attempt					865.34 (< 0.001)
No	21,261	83.5	65.2	34.8	
Yes	4193	16.5	41.0	59.0	
Current marijuana use					170.44 (< 0.001)
No	24,508	96.3	62.0	38.0	
Yes	946	3.7	40.9	59.1	
Truancy					391.59 (< 0.001)
No	18,694	73.4	64.8	35.2	
Yes	6760	26.6	51.2	48.8	
Close friends					0.08 (0.776)
No	2581	10.1	60.9	39.1	
Yes	22,873	89.9	61.2	38.8	
Peer support					134.46 (< 0.001)
No	17,310	68.0	58.8	41.2	
Yes	8144	32.0	66.4	33.6	
Parental or guardian supervision					26.28 (< 0.001)
No	14,519	57.0	59.8	40.2	
Yes	10,935	43.0	63.0	37.0	
Parental or guardian connectedness					108.60 (< 0.001)
No	15,215	59.8	58.6	41.4	
Yes	10,239	40.2	65.1	34.9	
Parental or guardian bonding					62.14 (< 0.001)
No	15,690	61.6	59.3	40.7	
Yes	9764	38.4	64.3	35.7	

Source: GSHS, 2010–2017

### Multivariable regression analysis of predictors of bullying victimisation among in-school adolescents in SSA

Table 3 presents the multivariable logistic regression results of the predictors of bullying victimization among the respondents in SSA. From the adjusted model, adolescents aged 15 years or older had lower odds for bullying victimization as against their counterparts aged 14 years or younger [aOR = 0.74, 95% CI = 0.69, 0.78]. Adolescents who felt lonely were more likely to be bullied compared to those who did not feel lonely [aOR = 1.66, 95% CI = 1.53, 1.80]. Also, adolescents with history of anxiety [aOR = 1.53, 95% CI = 1.41, 1.66], suicidal ideation [aOR = 1.28, 95% CI = 1.17, 1.39], suicidal attempt [aOR = 1.86, 95% CI = 1.72, 2.02], current use of marijuana [aOR = 1.59, 95% CI = 1.38, 1.84], truant at school [aOR = 1.43, 95% CI = 1.34, 1.52] were more likely to be victims of bullying. Adolescents who had peer support [aOR = 0.78, 95% CI = 0.73, 0.82], those with parental or guardian connectedness [aOR = 0.85, 95% CI = 0.80, 0.91], and those with parental or guardian bonding [aOR = 0.94, 95% CI = 0.88, 0.99] were less likely to be victims of bullying. Additionally, adolescents from Eswatini [aOR = 0.66, 95% CI = 0.58, 0.74], Mauritius [aOR = 0.41, 95% CI = 0.36, 0.46], and Tanzania [aOR = 0.46, 95% CI = 0.41, 0.53] were less likely to be bullied. The odds of bullying victimization were higher among adolescents from Ghana [aOR = 1.34, 95% CI = 1.18, 1.49], Mozambique [aOR = 1.18, 95% CI = 1.03, 1.37], and Sierra Leone [aOR = 1.64, 95% CI = 1.45, 1.86].

**Table 3:** Multivariable regression analysis of predictors of bullying victimisation among in-school adolescents in sub-Saharan Africa

Variable	Bullying victimisation	
	Model I aOR (95% CI) ***p***-value	Model II aOR (95% CI) ***p***-value
Age		
14 years or younger	1.0	1.0
15 years or older	0.84 (0.79, 0.88) < 0.001	0.74 (0.69, 0.78) < 0.001
Felt lonely		
No	1.0	1.0
Yes	1.75 (1.62, 1.90) < 0.001	1.66 (1.53, 1.80) < 0.001
Anxiety		
No	1.0	1.0
Yes	1.64 (0.51, 1.78) < 0.001	1.53 (1.41, 1.66) < 0.001
Suicidal ideation		
No	1.0	1.0
Yes	1.20 (1.10, 1.31) < 0.001	1.28 (1.17, 1.39) < 0.001
Suicidal plan		
No	1.0	1.0
Yes	1.11 (1.02, 1.21) 0.012	1.07 (0.98, 1.16) 0.118
Suicidal attempt		
No	1.0	1.0
Yes	1.99 (1.84, 2.16) < 0.001	1.86 (1.72, 2.02) < 0.001
Current marijuana use		
No	1.0	1.0
Yes	1.48 (1.29, 1.71) < 0.001	1.59 (1.38, 1.84) < 0.001
Truancy		
No	1.0	1.0
Yes	1.51 (1.43, 1.61) < 0.001	1.43 (1.34, 1.52) < 0.001
Peer support		
No	1.0	1.0
Yes	0.76 (0.72, 0.81) < 0.001	0.78 (0.73, 0.82) < 0.001
Parental or guardian supervision		
No	1.0	1.0
Yes	1.01 (0.95, 1.07) 0.854	0.98 (0.93, 1.04) 0.604
Parental or guardian connectedness		
No	1.0	1.0
Yes	0.86 (0.81, 0.92) < 0.001	0.85 (0.80, 0.91) < 0.001
Parental or guardian bonding		
No	1.0	1.0
Yes	0.94 (0.89, 1.00) 0.056	0.94 (0.88, 0.99) 0.031
Country		
Benin		1.0
Eswatini		0.66 (0.58, 0.74) < 0.001
Ghana		1.34 (1.18, 1.49) < 0.001
Liberia		1.02 (0.88, 1.17) 0.826
Mauritania		1.08 (0.94, 1.24) 0.298
Mauritius		0.41 (0.36, 0.46) < 0.001
Mozambique		1.18 (1.03, 1.37) 0.020
Namibia		1.02 (0.92, 1.14) 0.683
Seychelles		0.96 (0.84, 1.09) 0.496
Sierra Leone		1.64 (1.45, 1.86) < 0.001
Tanzania		0.46 (0.41, 0.53) < 0.001
N	25,454	25,454
Pseudo R^2^	0.0541	0.0807

*AOR* Adjusted Odds Ratio, *CI* Confidence Interval, 1.0 = Reference category

## Discussion

The current study examined the prevalence and predictors of bullying victimisation among in-school adolescents in 11 sub-Saharan African countries using data from the GSHS. The study found a 38.8% prevalence of bullying victimisation among in-school adolescents in SSA. Age, loneliness, anxiety, suicidal ideation, suicidal attempt, current marijuana use, truancy, peer support, parental or guardian connectedness, and parental or guardian bonding were found as predictors of bullying victimisation. The prevalence of 38.8% noted in this study is lower than what was found in Nepal [2], Mozambique [3], Malawi [13] and Ghana [11], except Nigeria which recorded a prevalence of 16.3% [12]. Socio-cultural, contextual and socio-economic variations (e.g., income inequality and disparity in educational spending) in the sub-region could contribute to bullying victimisation. While Sierra Leone recorded the highest prevalence, Mauritius had the lowest prevalence rate of bullying victimisation. The Sierra Leone case is unsurprising because post-conflict dynamics (e.g., social exclusion through turbulent moments) could trigger compulsive behaviours in school-going adolescents who may still be having repetitive thoughts or images of all the aggressive behaviours during the conflict period in the country that they cannot control. Therefore, emotions (e.g., anger, anxiety) caused by these thoughts could trigger impulses or aggressive actions and other forms of antisocial and/ or violent behaviorurs such as bullying that are distressing to others school mates [36]. Given the high prevalence of bullying victimisation among in-school adolescents in SSA, there is an urgent need to overhaul, reassesse and further improve the existing interventions on bullying prevention in schools in SSA.

Similar to other studies [3, 15, 21, 34], adolescents aged 15 years or older had lower odds for bullying victimisation than those aged 14 years or younger. All things being equal, adolescents who are older have the physical strength and mental toughness to resist or protect themselves from being bullied [13, 15]. Alternatively, younger adolescents may lack the ability to effectively cope with physical or cognitive-emotional obstructions or conflicts at that young age of adolescence [3]. Based on the findings, it is necessary to implement anti-bullying preventive interventions (e.g., Rational Emotive Behavioral Education, [REBE]) in schools that target the protection of younger adolescents. There is also the need to reinforce coping skills in students at younger ages.

Corroborating other previous studies [2, 13, 21], adolescents who felt lonely were more likely to be bullied than their counterparts who did not feel lonely. From the maladaptive schema perspective, loneliness from rejection is quite relevant in the context of bullying victimisation, as previous research has identified that schemas like loneliness could happen as a consequence of victimisation at school [37–39]. According to Calvete and associates, adolescents who are rejected by peers and experience insults and humiliation can develop cognitions and feelings of loneliness that are characteristic of maladaptive schemas (e.g., feeling defective, rejected and believing that others will intentionally abuse them). Also, perpetrators might have impressions that loneliness of their victims suggests that they might be unprotected from being bullied, as a result, may predispose lonely adolescents to frequent bullying. Similarly, perpetrators may also have the conviction that lonely people may have been neglected by their peers because of bad deeds; hence, bullying them is a way of paying them back for their wrong doings. This finding necessitates that school authorities continuously improve already existing interventions (e.g., building social support networks to boost belongingness and acceptance) that eliminate loneliness in schools to prevent bullying. Such social support networks should be strengthened through the provision of continuous monitoring and supervision. Members of the social support networks should also be encouraged to play their active roles in helping students who go through loneliness.

The findings that adolescents with a history of anxiety, suicidal ideation, suicidal attempt, current use of marijuana and truant at school are more likely to be victims of bullying are consistent with previous studies [2, 11, 21, 34]. There is a connection between bullying victimisation and maladaptive behaviours (e.g., suicidal ideations and attempts, drug use, delinquency) through feelings of unwantedness [40]. Individuals who have been bullied, go on to under-value or belittle and/ or develop negative thoughts about themselves, their personality, and subsequent future behaviours [41]. For instance, adolescents who experience multiple forms of bullying victimisation may be at risk for adjustment problems, including internalising problems and externalising problems (e.g., social anxiety, depression, frequent with substance use) [32]. Again, adolescents who experience psychologically unstable behaviours as cited earlier may frequently have conflicts with potential bullies as retaliatory attitudes. Specifically, victims who use marijuana and are always truant at school may show high resistance to bullying, which may likewise increase their susceptibility of being bullied. This finding implies that behaviour modification interventions (REBE) should be instituted at schools, and those that are already in existence should be improved.

Akin to previous studies [13, 21], other results showed that adolescents who had peer support were less likely to be victims of bullying than those who did not. Research evidence has shown that peer witnesses’ responses are crucial towards inhibiting or fuelling bullying victimisation. According to Palladino et al. [41], formally assigning peers as educators’ (i.e., involving them in awareness creation) has been proven to be very effective in reducing bullying victimisation among school-going adolescents. Therefore, enhancing peer awareness, empathy and self-efficacy to support victimised peers could be part of anti-bullying programs in schools [42]. For instance, adolescents who have peer support may be seen as well-behaved individuals and may ward off any possible bullying behaviour from perpetrators. Similarly, those who receive peer support feel socially protected from bullying acts. Further studies to better understand the role peer support plays in alleviating bullying victimisation among adolescents in the school setting is encouraged.

Adolescents from Eswatini, Mauritius and Tanzania were less likely to be bullied compared to countries such as Ghana, Mozambique and Sierra Leone, which showed higher odds of bullying victimisation among in-school adolescents. These former cited countries which are found around the same geographical region (i.e., South-east Africa) have similar socio-cultural and socio-economic characteristics that perhaps reduce the likelihood of bullying victimisation among in-school adolescents. Research has already shown that economically unequal countries where there are income inequalities and disparities in educational spending by governments could trigger anti-social behaviors (e.g., physical fighting and other aggressive behaviors) among school-going youths [43]. Socio-cultural variations associated with the term “bullying” might also account for the current finding [44]. However, because the data used employed standardised definitions and recoding of some variables, it seems unlikely that this noted finding is an artifact of cultural heterogeneity and/ or measurement of variables on the GSHS questionnaire. More studies are therefore warranted to seek for better understanding of why adolescents are more vulnerable to bullying victimisation in some nations than in others.

### Strengths and limitations

This study should be considered with some strengths and limitations. First, the use of nationally-representative survey data forms the GSHS of eleven sub-Saharan African countries supports the accuracy and reliability of the findings. The use of questionnaires for the secondary data permitted the assessment of multiple factors associated with bullying victimisation. The large sample size selected using a systematic random procedure with a high response rate warrants generalizability of findings to other homogenous populations. However, this study has some limitations. First, the assessment of bullying victimisation was based on self-reports, hence may be subject to recall and social desirability bias. Due to the cross-sectional design nature of the survey data, the factors noted in this study are devoid of any causality and thus precludes robust interpretations of current associations. The studied determinants also excluded cultural and historical antecedents among selected sub-Saharan African countries that are likely to shape norms and attitudes related to adolescents’ bullying victimisation.

## Conclusions

Our findings suggest that age, loneliness, history of anxiety, suicidal ideation, suicidal attempt, and current use of marijuana are associated with increased risk of bullying victimisation among adolescents. These findings indicate the value of in-school indicators of bullying victimisation and reiterate the importance of understanding how multidimensional factors may influence negative behaviour. Given the long-term psychological, physical and emotional consequences of bullying victimisation on the health of in-school adolescents, understanding current estimation and predictive factors could help with timely identification and management. In curbing the situation in selected sub-Saharan African countries, designing and implementing proactive interventions (e.g., Positive Behavioral Interventions- Rational Emotive Behavioral Education, [REBE], peer educator network systems, substance use cessation therapy, face-face counselling sessions) in schools are required. Given that the current sample involved secondary data from only in-school adolescents, comparing measured variables on data from out of school adolescents in future studies could be quite interesting. Additional school-level research could also target which specific school-contextual factors (e.g., high student–teacher ratio, school size, teacher characteristics) could best predict higher rates of bullying victimisation and possibly draw causal associations.

## Supplementary Information

**Additional file 1: Table S1.** Links to questionnaires and Datasets. **Table S2.** Study variables.

## Data Availability

The datasets generated and/or analysed during the current study are available in the WHO’s website (https://www.who.int/teams/noncommunicable-diseases/surveillance/systems-tools/global-school-based-student-health-survey). The specific links to each country’s publicly available datasets has been provided in Table S1. The cleaned datasets used and/or analysed during the current study are available from the corresponding author on reasonable request.

## Abbreviations

aOR: Adjusted Odds Ratio
CDC: Centre for Disease Control and Prevention
CI: Confidence Interval
SSA: Sub-Saharan Africa
LMICs: Low- and middle-income countries (LMICs)
VIF: Variance Inflation Factor
GSHS: Global School-based Health Survey
WHO: World Health Organization
