# Supplementary material for: A multi-country analysis of the prevalence and factors associated with bullying victimisation among in-school adolescents in sub-Saharan Africa: evidence from the global school-based health survey
Source: BMC Psychiatry. 2021 Jul 1;21:325. doi: 10.1186/s12888-021-03337-5 (PMC8252267; doi:10.1186/s12888-021-03337-5)
Supplement: Supplementary file 1 — Additional file 1: Table S1. Links to questionnaires and Datasets. Table S2. Study variables. [file 12888_2021_3337_MOESM1_ESM.docx]

**Table S1: Links to questionnaires and Datasets**

| Country | Link to dataset | Link to questionnaire |
| --- | --- | --- |
| 1. Benin(2016) | <https://www.who.int/ncds/surveillance/gshs/BJH2016_GSHS_Data_public_use.dat> | <https://www.who.int/ncds/surveillance/gshs/BJH2016_GSHS_Data_public_use_codebook.pdf?ua=1> |
| 1. Eswatini(2013 | <https://www.who.int/chp/gshs/SZH2013_public_use.dat> | <https://www.who.int/chp/gshs/SZH2013_public_use_codebook.pdf?ua=1> |
| 1. Ghana(2012) | <https://www.who.int/chp/gshs/GHDH2012_public_use.dat> | <https://www.who.int/chp/gshs/2012_Ghana_GSHS_Questionnaire.pdf?ua=1> |
| 1. Liberia(2017) | <https://extranet.who.int/ncdsmicrodata/index.php/catalog/646/download/4628> | <https://extranet.who.int/ncdsmicrodata/index.php/catalog/646/download/4624> |
| 1. Mauritania(2010) | <https://www.who.int/chp/gshs/MJH2010_public_use.dat> | <https://www.who.int/chp/gshs/MJH2010_public_use_codebook.pdf?ua=1> |
| 1. Mauritius (2017) | <https://extranet.who.int/ncdsmicrodata/index.php/catalog/669/download/4823> | <https://extranet.who.int/ncdsmicrodata/index.php/catalog/669/download/4819> |
| 1. Mozambique(2015) | <https://www.who.int/chp/gshs/MZH2015_public_use.dat> | <https://www.who.int/chp/gshs/MZH2015_public_use_codebook.pdf?ua=1> |
| 1. Namibia(2013) | <https://www.who.int/chp/gshs/NBH2013_public_use.dat> | <https://www.who.int/chp/gshs/NBH2013_public_use_codebook.pdf?ua=1> |
| 1. Seychelles(2015) | <https://www.who.int/chp/gshs/SHH2015_public_use.dat> | <https://www.who.int/chp/gshs/SHH2015_public_use_codebook.pdf?ua=1> |
| 1. Sierra Leone(2017) | <https://extranet.who.int/ncdsmicrodata/index.php/catalog/772/download/5396> | <https://extranet.who.int/ncdsmicrodata/index.php/catalog/772/download/5392> |
| 1. Tanzania(2014) | <https://www.who.int/chp/gshs/TZH2014_public_use.dat> | <https://www.who.int/chp/gshs/TZH2014_public_use_codebook.pdf?ua=1> |

**Table S2. ﻿ Study variables**

| **Variables** | **Question** | **Response options and recoding** |
| --- | --- | --- |
| **Outcome variable** | | |
| Bullied | ﻿During the past 30 days, how were you bullied most often? | ﻿1 = 0 times; to 8 = 12 or more times (coded as 1 = 0; and 2–7 = 1) |
| **Explanatory variables** | | |
| Socio-demographic characteristics | | |
| Age | How old are you? | 1=12, 2=13, 3=14, 4=15, 5=16, 6=17, 7=18 years (coded as 0=1-14, 15-19) |
| Sex | What is your sex? | 1=male, 2=female (coded 2=0, 1=male) |
| Psychosocial environmental factors | | |
| Marijuana use | During the past 30 days, how many times have you used marijuana (also called wee, Jah, Indian hemp etc.. | 1=0 times; to 5=20 or more times (coded as 1=0; and 2-5=1) |
| Loneliness | ﻿During the past 12 months, how often have you felt lonely? | ﻿1=never, 2=rarely, 3= sometimes, 4 = most of the time to 5 = always (coded as 1-3 = 0; and 4- 5 = 1) |
| Truancy | ﻿During the past 30 days, on how many days did you miss classes or school without permission? | 1=0 days,2= 1or 2 days, 3=3 to 5 days, 4=6 to 9 days, 5= 10 or more (coded as 1=0; and 2-5=1) |
| Anxiety | ﻿During the past 12 months, how often have you been so worried about something that you could not sleep at night? | ﻿1 = never to 5 = always  (coded 1 - 3 = 0, 4 – 5 = 1**)** |
| Suicidal ideation | During the past 12 months, did you ever seriously consider attempting suicide?” | 1 = yes, 2 = no (coded 2 = 0; and 1 = 1) |
| Suicidal plan | During the past 12 months, did you make a plan about how you would attempt suicide? | 1 = yes, 2 = no (coded 2 = 0; and 1 = 1) |
| Suicidal attempt | During the past 12 months, how many times did you actually attempt suicide? | 1=0 times; to 5= 6 or more times (coded as 1=0; and 2-5=1) |
| Close friends | ﻿How many close friends do you have? | 1=0 to 4=3 or more (coded as 1=0; and 2-4=1) |
| Helpful  (Peer support) | ﻿During the past 30 days, how often were most of the students in your school kind and helpful? | 1=never, 2=Rarely, 3=sometimes, 4=most of the times, 5=always  (coded as 1-3 = 0; and 4–5 = 1) |
| Parents check homework (parental supervision) | ﻿During the past 30 days, how often did your parents or guardians check to see if your homework was done? | 1=never, 2=Rarely, 3=sometimes, 4=most of the times, 5=always  (coded as 1-3 = 0; and 4–5 = 1) |
| Understand problems  ﻿ (Parental  Connectedness | ﻿During the past 30 days, how often did your parents or guardians understand your problems and worries? | ﻿1=never, 2=Rarely, 3=sometimes, 4=most of the times, 5=always  (coded as 1-3 = 0; and 4–5 = 1) |
| Know what adolescent do free time (﻿Parental or guardian  Bonding) | ﻿During the past 30 days, how often did your parents or guardians really know what you were doing with your free time? | ﻿1=never, 2=Rarely, 3=sometimes, 4=most of the times, 5=always  (coded as 1-3= 0; and 4–5 = 1) |
